# Delayed primary closure of contaminated abdominal wall defects with non-crosslinked porcine acellular dermal matrix compared with conventional staged repair: a retrospective study

**DOI:** 10.1186/1752-1947-8-251

**Published:** 2014-07-11

**Authors:** Hans M Schardey, Francesca Di Cerbo, Thomas von Ahnen, Martin von Ahnen, Stefan Schopf

**Affiliations:** 1 Department of General, Visceral and Vascular Surgery, Agatharied Academic Teaching Hospital of the Ludwig Maximilians University, Norbert Kerkel Platz, D 83734 Agatharied, Germany

**Keywords:** Contaminated hernia repair, Strattice, Cost, Porcine-derived acellular dermal matrix

## Abstract

**Introduction:**

Synthetic mesh has been used traditionally to repair abdominal wall defects, but its use is limited in the case of bacterial contamination. New biological materials are now being used successfully for delayed primary closure of contaminated abdominal wall defects. The costs of biological materials may prevent surgeons from using them. We compared the conventional staged repair of contaminated abdominal wall defects with a single-stage procedure using a non-crosslinked porcine acellular dermal matrix.

**Methods:**

A total of 14 cases with Grade 3 contaminated abdominal wall defects underwent delayed primary closure of the abdomen using a non-crosslinked porcine acellular dermal matrix (Strattice™ Reconstructive Tissue Matrix, LifeCell Corp., Branchburg, NJ, USA). The results were compared with a group of 14 patients who had received conventional treatment for the repair of contaminated abdominal wall defects comprising a staged repair during two separate hospital admissions employing synthetic mesh. Treatment modalities, outcomes, and costs were compared.

**Results:**

In all cases treated with delayed primary closure employing non-crosslinked porcine acellular dermal matrix, there were no complications related to its use. Two patients died due to unrelated events. Although treatment costs were estimated to be similar in the two groups, the patients treated with porcine acellular dermal matrix spent less time as an inpatient than those receiving conventional two-stage repair.

**Conclusions:**

Delayed primary closure of contaminated abdominal wall defects using a non-crosslinked porcine acellular dermal matrix may be a suitable alternative to conventional staged repair. In our patients, it resulted in early restoration of abdominal wall function and shorter hospitalization. The costs for treating contaminated abdominal wall defects using porcine acellular dermal matrix during a single hospital admission were not higher than costs for conventional two-stage repair. Further randomized studies are needed to expand upon these findings.

## Introduction

The reconstruction of simple abdominal wall defects can be challenging, but when the defects are contaminated, infected, very large, or have recurred, a durable functional reconstruction can be difficult to achieve. The most common abdominal wall defects are incisional or recurring incisional hernias, but defects may also result from trauma, extensive tumor resection, or necrotizing infection in the abdominal wall. In 2004, a study recorded a failure rate of 75 percent for suture repairs of simple incisional hernias after an observation period of 10 years [1]. The results of a prospective, randomized, multicenter trial in 2000 showed that incisional hernias should always be repaired with a synthetic mesh [2]. However, failure rates of repairs employing synthetic mesh are quite high, with one study showing a failure rate of 23 percent after three years and 35 percent after 10 years [3]. The authors also reported that after each repair, the risk of failure increased [3]. The main reason for failure seems to be poor technique. Another very serious reason for the failure seems to be infection, which has been identified by multivariate analysis to be a highly significant factor for the recurrence of incisional hernias [2].

Even when placed into sterile tissue, synthetic mesh may shrink, migrate, or cause adhesions, fistulae, and pain as a result of ongoing chronic inflammation [4-6]. There is evidence that synthetic mesh should not be used in the presence of bacterial contamination or infection [7]. Mesh infection may lead to the formation of fistulae or even mesh extrusion, and can only be treated by radical removal of the mesh. The results of a meta-analysis revealed that after an initial successful implantation of conventional heavyweight synthetic mesh into contaminated abdominal wall defects, 90 percent had to be explanted [8]. Today, the evidence is growing, suggesting that modern lightweight meshes behave more favorably in the face of contamination. A recent, a retrospective, single-institution study of the use of synthetic mesh in contaminated fields showed no increased risk of infection or gastrointestinal fistulae [9]. However, this approach remains controversial, and prospective randomized studies are needed to provide further evidence.

In Europe, the traditional strategy used to close an infected or contaminated abdominal wall defect is a two-stage procedure requiring two separate hospital admissions. The principles are to control the intra-abdominal complication and then deal with the infection in the plane of the abdominal wall. It may be necessary to reduce the bioburden, for example by sharp debridement and the use of negative pressure wound therapy (NPWT). In some cases the patient can be managed with a laparostomy alone. In some cases the bowel is allowed to granulate with or without the use of a resorbable mesh. After a short period, the defect is covered with a split-thickness skin graft or the skin is closed over the defect and the patient is discharged. Without a functional abdominal wall, the patient has a hernia by definition. This requires further surgical intervention three to 18 months following the initial procedure to repair the hernia as part of a planned abdominal reconstruction.

There have been a number of reports [10] on the use of biological materials derived from human or animal sources for the delayed primary closure of these high-risk contaminated or infected abdominal wall defects. Since 2009, a structurally intact, non-crosslinked porcine acellular dermal matrix (PADM) has been available in Europe and marketed under the trade name Strattice™ Reconstructive Tissue Matrix (LifeCell Corp., Branchburg, NJ, USA). The biological characteristics of this PADM may facilitate tissue regeneration by encouraging revascularization and repopulation of host cells, while minimizing the risk of encapsulation and fibrotic tissue formation [11].

The aim of this article is to report our experience using PADM for the delayed primary repair of contaminated abdominal wall defects and to compare these cases to a historic, previously treated group who had received conventional treatment of their contaminated abdominal wall defect during their initial hospital admission and a planned hernia repair with lightweight synthetic mesh at a later stage during a second hospital admission.

## Results

The male-to-female ratio in the two study populations was similar. The average age at the time of the repair was 69.1 years (range 41 to 88 years) in Group 1 and 66.5 years (range 47 to 82 years) in Group 2. Both groups included patients with minor (perforated appendicitis, cholecystitis, stoma reversal) and major (intestinal perforation, major colorectal procedures) problems on admission (Table 1). Emergency surgery was required in eight patients in Group 1 and seven in Group 2. The average body mass index (BMI) was five points higher in Group 2 (30kg/m^2^) compared with Group 1 (25kg/m^2^); other comorbidities such as diabetes, obesity, or smoking were present in both groups, and many patients had multiple risk factors for infection (Table 2).

**Table 1:** Patient diagnosis on admission

	***Group 1***	***Group 2***
Perforated appendicitis	1	3
Acute cholecystitis	1	
Incisional hernia with fistula	1	
Infection of synthetic mesh	1	
Vascular procedure		1
Ileus +/− intra-abdominal abscess		2
Planned colorectal procedure	3	3
Crohn’s disease	1	1
Diverticulitis/intestinal perforation	6	4

**Table 2:** Comorbid conditions increasing the risk for wound infection

	***Group 1***	***Group 2***
Average body mass index	25	30
Obese >30kg/m^2^	1	3
Smoker		2
Diabetes	2	1
History of soft tissue infection	2	3
Hypoxemia	1	1
Renal insufficiency	1	0
Corticosteroid therapy	1	1
Benign prostate hyperplasia		2

The extent of surgery carried out was comparable in both groups. Nine patients required bowel resection in each group (Table 3). The postoperative course for both groups is shown in Table 4. Seven patients in Group 1 and eight patients in Group 2 required treatment of peritonitis. Nine patients in Group 1 and 11 patients in Group 2 had an abdominal wall infection. Swabs from abdominal wall defects were cultured at initial surgery and a wide spectrum of bacteria were detected in both groups (Table 5). Both groups were comparable regarding preoperative American Society of Anesthesiologists (ASA) score, average number of days of intensive care treatment, number of surgical procedures, and number of NPWT dressing changes.

**Table 3:** Main operative procedures carried out

	***Group 1***	***Group 2***
Appendectomy	1	3
Cholecystectomy	1	
Bowel resection	9	9
Excision of infected mesh	1	
Incisional hernia repair	1	
Parastomal hernia repair	1	
Bifurcated aortic graft		1
Adhesiolysis/drainage of abscess		1

**Table 4:** Postoperative course

	***Group 1***	***Group 2***
Abdominal wall infection as a complication of primary surgery	9	11
Peritonitis	7	8
Preoperative ASA score	1.9	2.1
Mean days in ICU	7.6 (range 0–41)	9.3 (range 0–45)
Mean number of surgical procedures	3.0 (range 1–9)	3.2 (range 1–10)
Mean number of NPWT dressing changes	1.2 (range 0–3 )	1.0 (range 0–7)
Mean number of days in-hospital stay	27.2 (range 9–70)	26.1 (range 7–70)
Hernia on discharge first admission	0	4
Removal of mesh or PADM	0	0
Rate of recurrence at one year	1 (7.1%)	1 (7.1%)

ASA, American Society of Anesthesiologists; NPWT, negative pressure wound therapy; PADM, porcine acellular dermal matrix; ICU, intensive care unit.

**Table 5:** Micro-organisms cultured from abdominal wound swabs

	***Group 1***	***Group 2***
Streptococcus	1	1
*Staphylococcus epidermidis*	1	1
*Staphylococcus aureus*	1	1
*Enterococcus*	1	1
*Escherichia coli*	2	1
Klebsiella	1	2
*Proteus mirabilis*	1	
*Citrobacter freudii*	1	1
*Morganella morgagni*		1
*Seratia marcenscens*		1
No growth	2	2

**Figure 1:**
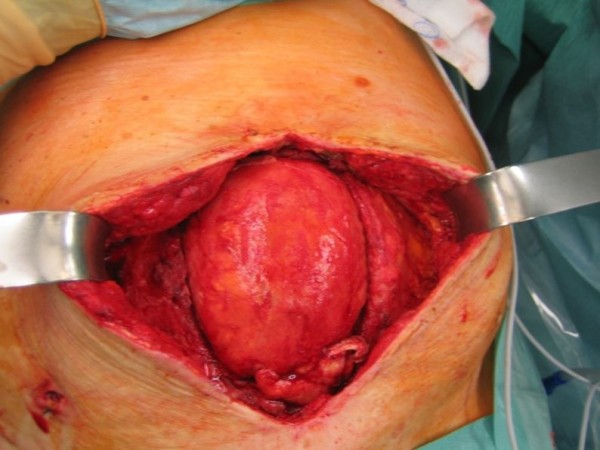
Open abdomen in a 78-year-old woman with a frozen abdomen and retracted fascial edges.

**Figure 2:**
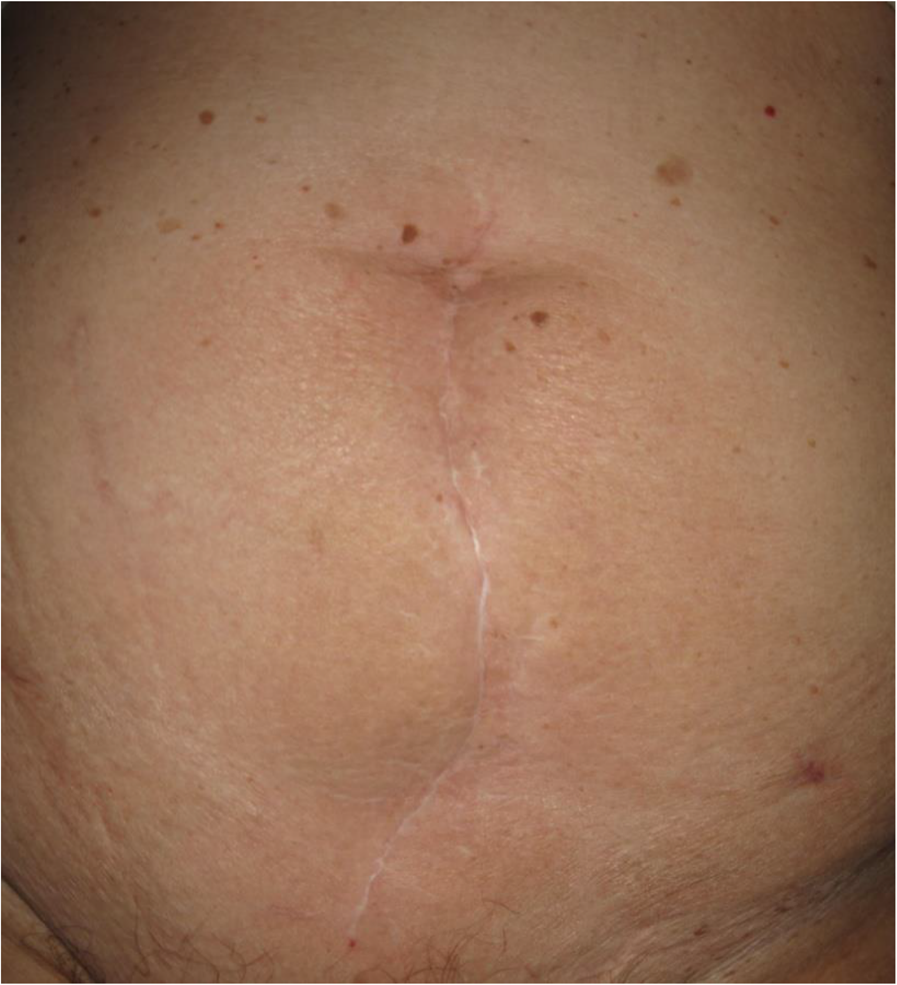
**Results 12 months after surgery using a single-stage procedure employing component separation to restore the midline and reinforce it with porcine acellular dermal matrix in a 78-year-old woman with an open abdomen (the same patient as in Figure**1**).**

**Figure 3:**
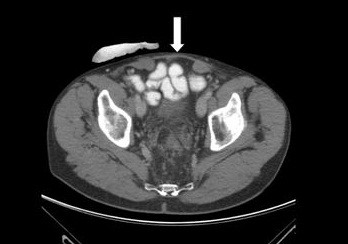
Computed tomography scan of a patient with cancer showing the non-crosslinked porcine acellular dermal matrix (arrow) nine months after implantation.

**Figure 4:**
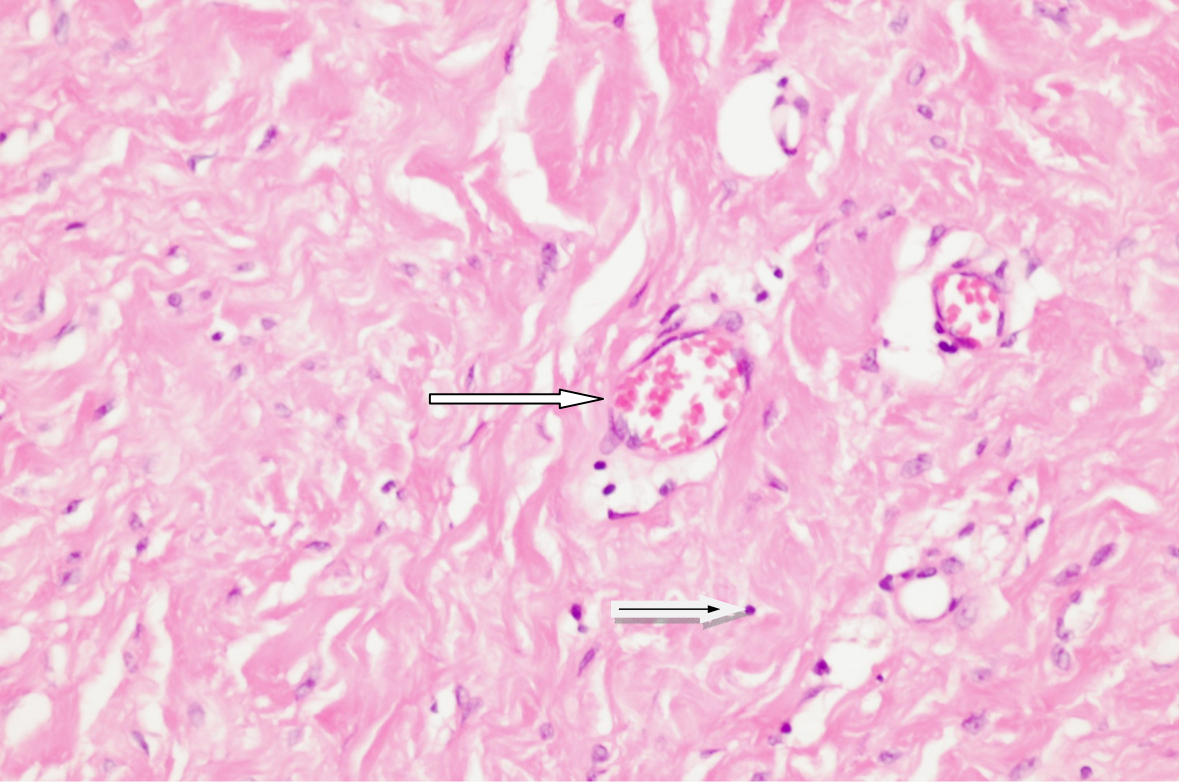
**Histologic section (hematoxylin & eosin staining) of non-crosslinked porcine acellular dermal matrix biopsy nine months after implantation into a colorectal cancer patient under chemotherapy.** Fibroblasts (black arrow in white arrow) and blood vessels (white arrow) are shown within the scaffold.

Repair of Grade 3 contaminated abdominal wall defects with PADM was successful in all cases in Group 1 at a median of 20 days after admission (Table 6). Median size of the abdominal wall defects in Group 1 was 75cm^2^ (range 45 to 250cm^2^). Median PADM size was 112cm^2^ (range 112 to 320cm^2^). There was one onlay repair of an incarcerated parastomal hernia. Eleven patients received intraperitoneal (three patients) or retrorectus sublay (eight patients) placement of PADM beneath the muscle. Reconstruction of the midline was the goal in all midline incisions and was carried out using component separation to advance the rectus complex medially in two patients with severely retracted fascial edges in the presence of a frozen abdomen (Figures 1 and 2). In non-midline incisions, the fascial edges were also brought together. In one case of a right upper-quadrant incision, PADM was placed into an intraperitoneal sublay position, and in two cases of Pfannenstiel incisions, PADM was placed beneath the ventral sheath of the rectus muscle.The approximation of the fascial edges could not be achieved in one patient due to an extensive full-thickness excision of the fascia, muscle, and skin. In addition, there was insufficient overlap due to the unavailability of an adequately sized piece of PADM at the time of surgery, resulting in a failure to achieve a sufficiently wide overlap when covering the defect. This patient underwent the same repair procedure twice, the first time below the umbilicus and the second time above it. The indication for surgery each time was the removal of one or more metastases. At re-operation nine months after the first repair, adequate mechanical stability of the abdominal wall remained. A computed tomography (CT) scan showed the PADM in place and physically closing the defect (Figure 3). A biopsy from the middle of the PADM nine months after implantation showed good ingrowth of host fibroblast and development of blood vessels in the PADM (Figure 4). However, at 12 months the patient developed ascites due to tumor progression and a recurrence was detected in the area of the midline repair below the umbilicus where the bridging had been carried out.

**Table 6:** Treatment costs based on treatment time and procedures

	***Group 1***	***Group 2***
Median time to abdominal wall repair	20 days (range 0–58 days)	352 days (range 30–3100 days)
Mean costs for initial hospital admission	€21,542 (range 2871–63,603)	€20,089 (range 2300–60,962)
Mean costs of porcine acellular dermal matrix Group 1 or synthetic mesh Group 2	€2353 (range 1760–8000)	€450 (range 180–720)
Mean costs of staged hernia repair (with second hospital stay)		€3180 (range 2300–7427)
Mean in-hospital days on readmission for hernia repair		6.9 (range 3–10)

There were two intraoperative small bowel lesions in Group 1 patients during the repair that did not lead to postoperative infection. One patient treated for colon necrosis with peritonitis developed a wound infection involving the PADM that resolved without PADM removal. In one patient, a postoperative seroma was treated surgically three weeks after repair with PADM. Another patient on oral anticoagulants suffered a hemorrhage 10 days after surgery (Table 7).

**Table 7:** Surgical site occurrences after porcine acellular dermal matrix or mesh placement

	***Group 1***	***Group 2***
Deep wound infection	1	0
Seroma formation	1	2
Hematoma	1	1

One 78-year-old patient died from pneumonia three weeks after retrorectus sublay repair of a burst abdomen following emergency right colectomy for ischemic colon necrosis. One 88-year-old patient was admitted for incarcerated and perforated bowel in a parastomal hernia and died from persisting systemic sepsis. One 80-year-old patient died four months after hernia repair during cardiac surgery for four valve replacements. At that time, there was no sign of recurrence in this patient. In all three cases, local wound healing of the hernia repair had been uneventful.

In Group 2, the repair of Grade 3 contaminated abdominal wall defects during the first hospital admission was carried out by direct suture or by simply closing the skin over the bowel, as was done in four patients. These four patients left the hospital with a hernia by definition. At a median of approximately one year later (Table 6), patients presented with hernia defects measuring a median of 150cm^2^ (range 15 to 400cm^2^). In 10 patients, fascial closure was achieved without special measures. Component separation was used twice to accomplish midline closure. Synthetic mesh with a median size of 450cm^2^ (range 150 to 600cm^2^) was employed for the repair. Primilene™ (Braun Melsungen, Melsungen, Germany) was used nine times, Vipro II™ (Ethicon, Norderstedt, Germany) was used twice, and TiMesh™ (Pfm Medical AG, Cologne, Germany) was used three times. An onlay repair was done in one patient, and in another the mesh was placed between the internal and the external oblique muscle. In the other 12 patients, a retrorectus sublay repair was carried out. During the postoperative course, one patient developed a hemorrhage and two patients developed seromas. There were no mesh-related complications in Group 2. All complications were treated surgically. None of the patients required removal of the mesh (Table 7).

Group 1 patients remained in the hospital a mean of 27.2 days, six days less than the combined stays of Group 2 patients (mean of 33 days; 26.1 days first admission plus 6.9 days second admission).

The median time between infection control and the definitive abdominal wall repair was quite different for the two study groups: 20 days (range 0 to 58 days) after primary surgery in Group 1 and 352 days (range 70 to 3100 days) in Group 2 (Table 6). This was the time that passed before patients in Group 2 were readmitted to the hospital for hernia repair with synthetic mesh as part of their planned two-stage procedure.

All surviving Group 1 patients (78.5 percent) were clinically examined one year after hernia repair. There was one recurrence after one year, resulting in a recurrence rate of 7.1 percent. All surviving Group 2 patients (85.0 percent) were clinically examined a mean of six years (range two to 10 years) after hernia repair. No hernia recurrences were detected. There was one repair for recurrence one year after the staged repair in Group 2 patients amounting to a recurrence rate of 7.1 percent for the entire group, even though the duration of the observation period was longer for this group.

Using German diagnosis-related group (DRG) payment rates, average hospital reimbursement was €21,542 in Group 1 and €20,089 in Group 2 for the first hospital stay. There was an additional sum of €3180 for the planned hernia repair carried out during the second hospital admission in Group 2. In Group 1, there was a mean sum of €2353 for the purchase of the PADM and in Group 2 a mean sum of €450 for the purchase of the synthetic mesh. In both groups, the materials used for reconstruction were considered to be included in the DRG and not covered by separate reimbursements to the hospital from insurance companies. The overall costs in both groups were very similar and not increased by the use of PADM.

## Discussion

The results of this small, nonrandomized clinical study suggest that the repair of contaminated abdominal wall defects can either be accomplished with a staged procedure or by delayed primary closure of the abdomen using PADM. In this series, both techniques were successful at one year after final repair, had acceptable complication rates, and had recurrence rates of approximately 7 percent. Despite the higher price of the PADM, the costs of delayed primary closure of the abdomen using PADM do not seem to be higher than the staged procedure with synthetic mesh. The treatment strategy of closing the abdomen with reinforcement of the fascial suture line with PADM during a single admission led to an early restoration of the integrity of the abdominal wall, saved in-hospital days, and eliminated the need for a second costly surgical procedure.

We started to use PADM for abdominal wall reconstruction because, in the United States, such materials have been in extensive use for more than a decade [12]. These biologic tissue matrices were mostly used in contaminated fields, which has allowed for a one-stage repair with no or little subsequent matrix removal [10,12,13]. Unfortunately, long-term results are still not available. Kissane and Itani [12] point out that ventral incisional hernia repair with these matrices continues to be plagued by a high recurrence rate and complications. They also suggest that prospective randomized trials are needed to properly direct practice in the use of these matrices and evaluate their ultimate value. Recently, additional experimental [14] and clinical evidence [15] has appeared supporting the use of large-pore synthetic mesh in contaminated surgical fields. Slater and coworkers [16] carried out an extensive review of clinical trials of biological matrices and synthetic mesh. They concluded that: ‘In view of the current evidence, biologic grafts have similar results to synthetic mesh or autologous repair in either clean, contaminated, or complicated ventral hernia repair’. Nonetheless, guidelines for the use of synthetic material in contaminated fields are not yet available, while PADM is made for use in exactly that particular clinical setting [10,12,13]. The authors consider it to be an advantage to have a material that can be used to reinforce the repair of a contaminated abdominal wall defect, does not have to be removed in the case of infection, and causes no harm to the bowel either by aggressive adhesion formation or fistula development.

### Placement of the matrix

Key considerations for a successful outcome include the ability to restore the structural as well as the functional anatomy of the abdominal wall. This can be achieved using the biological repair material in a sublay position (either retrorectus or intraperitoneal) to reinforce the reconstruction [13,17]. Bridging of defects by fixing the matrix to the edges of the wound should be avoided and is associated with a high failure rate [17,18]. The one failure we observed was in a patient with a matrix bridging the defect. Due to the small number of patients, it is not possible to draw valid conclusions, but we had the impression that these patients tolerated the clearly less-invasive intraperitoneal placement of the PADM better than placement in the retrorectus sublay position. In the nonrandomized Repair of Infected Contaminated Hernias (RICH) study, 60 percent of the PADM was placed in the intraperitoneal underlay position [10]. Further randomized trials are needed to confirm the optimal placement of the PADM.

### Timing of surgery

Repair of complex abdominal defects is also technically quite demanding, especially when the repair is undertaken 10 to 15 days after the primary surgery. Adhesions may be severe, and a functional closure is often difficult to achieve at this stage. These difficulties were illustrated by the occurrence of small bowel injuries in two patients in Group 1. Nonetheless, the postoperative course in both patients was uneventful. However, the overall wound event rate was low, with the occurrence of wound infection in only one patient in Group 1 (7 percent).

Results from the multicenter RICH study [10] reported a wound event rate of 66 percent and an infection rate of 30 percent at 24 months. One of many differences between the current study and the RICH study is that single-stage repairs were carried out almost exclusively in the latter, which means that patients were admitted, the wound was cleaned and the PADM (Strattice™) was implanted at the end of the procedure. This was true not only in the clean-contaminated cases (49 percent) but even in the contaminated (49 percent) and dirty cases (2 percent). In contrast to this in the present series, all wounds in Group 1 were cleaned and conditioned with NPWT to the point where granulation tissue covered the surface, before PADM was used. This converted Grade 4 hernias to Grade 3 hernias (that is, those that ‘may be potentially contaminated due to previous wound infection’) [13]. The process of cleaning the wound to control the focus of infection prolonged the time to closure (mean 20 days) and allowed for the formation of adhesions. The intention had been to make the final repair safer by reducing the risk for infection, but this unintentionally increased adhesions and the risk for bowel injury and associated contamination. In addition, the duration of in-hospital stay and procedural costs increased. By gaining more experience with PADM, the approach practiced in the RICH study may be adopted in more cases in order to save costs.

In both the RICH study and in our series, it was not necessary to remove the PADM during the treatment of surgical-site occurrences. However, the rate of recurrence in the RICH study was 19 percent after one year and 28 percent after two years [10]. In the RICH study, large Grade 3 and 4 hernias [12] were treated in patients of whom 64 percent had undergone multiple hernia repairs, one third had experienced previous wound infections, and a quarter had a BMI greater than 30kg/m^2^. Despite the fact that these were high-risk hernia patients, the rate of recurrence may be related to the surgical strategy and to the high rate of postoperative wound events. And, of course, it is necessary to find out what really happens to PADM in the presence of infection.

### Choice of biological repair materials

Biological repair materials are a diverse and expanding class with a wide range of different properties. There are specific characteristics that are thought to contribute to successful use, especially in the setting of contamination or low-grade infection [13]. These properties include an intact extracellular matrix and the ability to support tissue regeneration through revascularization and cell repopulation in a clinically relevant timeframe. It has been hypothesized that resistance to infection for some biological repair materials may be related to the ingrowth of cells and vasculature [19]. A number of animal studies have shown that some crosslinking processes may damage the extracellular matrix, which may have a negative impact on the host response to the repair material [20] and prevent ingrowth of fibroblasts into the matrix [21]. Further research supports the theory that the ability of the immune system to recognize the matrix as ‘self’ is important for an optimal host response as a negative or poor recognition may result in absorption or encapsulation of the matrix [20,22]. An *in vitro* study has shown that a non-crosslinked tissue matrix may elicit a more moderate mononuclear cell response than crosslinked matrices, suggesting improved integration into host tissue [23].

For the purposes of this study, Strattice™ was selected for the repair of contaminated abdominal wall defects. It is a non-crosslinked PADM and has shown good results in experimental studies in nonhuman primates [11]. A CT scan at nine months post-surgery in a 71-year-old patient who had undergone delayed primary closure with Strattice™ in our series demonstrated the physical presence of the implanted matrix (Figure 3), while histological examination showed fibroblasts and vascular ingrowth (Figure 4).

### Selection process and outcomes

The groups were quite similar with respect to demographics, type of planned or emergency surgery, and postoperative complications. Patients from Group 1 were chosen due to the fact that the PADM had been used to repair the contaminated abdominal wall defect. The historical control group (Group 2) was selected on the basis of having had a staged repair of a Grade 3 [13] contaminated abdominal wall hernia, meaning that they were admitted to the hospital twice. Simple suture repair had been carried out in most of them in the presence of contamination. This strategy may result in a high rate of incisional hernia, reaching 69 percent after 10 years in some studies [24]. The duration between primary surgery for infection and final repair of the hernia averaged one year. This was one of the major differences between the two groups. Even though we do not have systematic quality-of-life data of our patients, it is well known that incisional hernias are a source of discomfort and can limit physical ability.

In Group 1, all surviving patients left the hospital with a reconstructed and stable abdominal wall approximately 20 days after initial treatment for infection. This is a major advantage. Early functional restitution of the abdominal wall was important to these patients, as evidenced by patient reports upon follow-up examinations. One year results were good (Figure 2), with only one recurrence likely due to poor technique (that is, bridging the defect with insufficient overlap [17,18]). There were two deaths in Group 1, one due to persistent sepsis after bowel perforation, the other due to pneumonia. Both deaths were unrelated to the PADM.

### Comparison of costs

The in-hospital costs of the initial hospital admission for both groups were similar as calculated according to the German DRG system (Table 6). However, in Group 2 patients treated with the staged repair, there was approximately one year between procedures and there is no documentation of the costs incurred during this period.

The costs of the incisional hernia repair during the second admission in Group 2 patients, including adjustment for comorbidities and complications as well as the in-hospital stay for an average of 6.9 days, amounted to a mean cost of €3180 (German DRG). These costs were higher than the mean cost of €2353 per patient for the PADM used to restore the functional and anatomical integrity of the abdominal wall in Group 1 patients during the initial hospital stay. The PADM size used in the majority of cases was 16×7cm, which represented a cost savings compared with the larger 20×20cm size used in 86 percent of patients in the RICH Study [10]. The costs for synthetic mesh or biologic matrices are not reimbursed separately by German health insurance companies.

### Limitations

The limitations of this report are its retrospective, single-institutional design, the selection of a historic control group, modest sample size, and a relatively short follow-up period in these high-risk hernia patients.

## Conclusions

The use of PADM is a new concept in medicine and offers a means to treat contaminated abdominal wall defects as well as defects at high risk for infection. The results of this study suggest on short-term observation that delayed primary closure using non-crosslinked PADM provides an equally safe and effective alternative to conventional staged repair of Grade 3 contaminated abdominal wall defects. PADM may offer faster recovery times and result in earlier restoration of abdominal wall function, thus reducing overall duration of physical impairment without increasing the costs of treatment, despite the cost of the biological scaffold. The reduction in the number of admissions and of in-hospital days achievable by using PADM may have a greater impact on costs in other health-care systems outside of Germany. Randomized trials are needed clarify these open questions.

## Methods

Under the approved Ludwig Maximilians University (LMU) of Munich ethics committee protocol, records of patients with contaminated abdominal wall defects treated at the Department of Surgery at Agatharied Academic Teaching Hospital of the LMU were reviewed retrospectively. The hospital database was searched using the Operations and Procedures (OPC) and International Statistical Classification of Diseases and Related Health Problems (ICD) codes for incisional hernias, burst abdomen, emergency surgery for perforated bowel, peritonitis, and abdominal wall infection documented between 1999 and 2010. Classification of the abdominal defect was made according to the Ventral Hernia Working Group (VHWG) recommendations [13] (Table 8) from the United States, where the use of biologic matrices is more common.

**Table 8:** Ventral Hernia Working Group assessment of incisional ventral hernias for risk of surgical site occurrences: definitions of grades

**Grade 1**	**Grade 2**	**Grade 3**	**Grade 4**
*Low risk of surgical-site occurrences*			*High risk of surgical-site occurrences*
No comorbidities	Patient has comorbidities, for example, smoker, obese, diabetic, immunosuppressed, chronic obstructive pulmonary disease	Wounds that are potentially contaminated due to a previous wound infection, presence of a stoma, or violation of the gastrointestinal tract	Wounds with active infection, such as septic dehiscence or the presence of an infected synthetic mesh
No history of wound infection	No evidence of wound contamination or active infection		
No evidence of contamination			

Adapted with permission from *Surgery*, 148, Breuing K, Butler CE, Ferzoco S, *et al.* Incisional ventral hernias: review of the literature and recommendations regarding the grading and technique of repair, 544–558, ©2010 [13].

In Group 1, 13 consecutive patients with 14 Grade 3 abdominal wall hernias had delayed primary closure of the abdominal wall defect using non-crosslinked PADM during a single hospital admission (during the study period, one patient was operated on twice due to tumor progression, both times using the same material and technique). In Group 2, 14 patients with abdominal wall defects classified as Grade 3 abdominal wall hernias during initial hospital admission were selected from the database. Patients in Group 2 received conventional two-stage repair of an abdominal wall defect (that is, treatment of the contaminated abdominal wall during the first hospital admission and an incisional hernia repair with synthetic mesh during the second hospital admission). All patients in both groups met one or more criteria for classification as a Grade 3 hernia (Table 9). In purulent infection, as described for Grade 4 hernias, no reconstruction was carried out before the wound was converted to a Grade 3 hernia using debridement, washout, and NPWT.

**Table 9:** Factors defining classification of Grade 3 contaminated abdominal wall hernias^*^

	***Group 1***	***Group 2***
Violation of the intestinal tract	10	11
Stoma present	8	7
History of mesh infection	1	0
Existing open wound	8	8

^*^Some patients presented with more than one factor.

The clinical course, facility costs, and patient outcomes were compared between groups. Demographics, comorbidities, BMI, ASA score, surgical notes, complications, and follow-up data were evaluated. Surgical data included date of surgery, indications for surgery, type of surgery (emergency or planned), fascial defect location, characteristics, defect size, mesh type and size, and number of surgical procedures.

### Surgical control of intra-abdominal and abdominal wall infection

Intra-abdominal infections resulting from intestinal perforations or other complications in both groups were treated by suturing, resection, or by creating diversion colostomies or ileostomies. The creation of high-risk anastomoses was avoided in most cases. In addition, an abdominal washout was included as a standard procedure. Relaparotomies and additional washouts were carried out when indicated. In severe cases, intra-abdominal NPWT was used to temporarily close the abdomen.

Sharp debridement of devitalized or infected tissue to reduce the wound bioburden was carried out at the level of the abdominal wall. Pulse lavage was applied in six cases (three in each group) to clean contaminated tissue. The majority of cases (n=20) received NPWT to the wound only until healthy granulation tissue covered the wound surface prior to closure.

### Group 1: delayed primary closure with PADM during one hospital admission

Fascial closure using a sublay reinforcement with PADM was performed in 13 cases. One patient underwent an emergency parastomal hernia repair using PADM with an onlay technique to reinforce the reconstruction. PADM was fixed to fascial structures with permanent Prolene size 0 sutures in all cases. PADM was placed under moderate tension with at least 3cm overlap beyond the fascial margins. This preset tension was used to minimize the stress to the fascial suture line [13]. Component separation technique, as described by Ramirez *et al.*[25], was performed to achieve a functional reconstruction of the linea alba. PADM was used in all 14 procedures.

### Group 2: staged repair of contaminated abdominal wall defects during two hospital admissions

Group 2 patients had undergone treatment to control intra-abdominal complications and infection of the abdominal wall. Four patients had been discharged with an abdominal wall defect using a skin-only repair to close the abdomen; in 10 patients, a hernia developed after a contaminated Grade 3 abdominal wall defect had been closed by suture repair. Patients were readmitted to the hospital for hernia repair with synthetic mesh several months after their first admission. Midline closure was the goal, with use of component separation as necessary.

## Consent

Written informed consent was obtained from the patients for publication of this case report and any accompanying images. A copy of the written consent is available for review by the Editor-in-Chief of this journal.

## Abbreviations

ASA: American Society of Anesthesiologists; BMI: body mass index; CT: computed tomography; DRG: diagnosis-related groups; ICD: International Statistical Classification of Diseases and Related Health Problems; LMU: Ludwig Maximilians University; NPWT: negative pressure wound therapy; OPS: Operations and Procedures; PADM: porcine acellular dermal matrix; RICH: Repair of Infected or Contaminated Hernias Study; VHWG: Ventral Hernia Working Group.

## Competing interests

SHM has been a paid speaker at LifeCell Corporation events. All other authors declare that they have no competing interests.

## Authors’ contributions

HMS conceived of the study and drafted the manuscript. FDC collected the data. TvA filed the application to the ethics committee and participated in the data collection. MvA participated in the design of the study, data analysis and presentation, and translated the article. SS participated in the study design and coordination and helped to draft the manuscript. All authors read and approved the final manuscript.
